# Novel Methods to Mobilize, Isolate, and Expand Mesenchymal Stem Cells

**DOI:** 10.3390/ijms22115728

**Published:** 2021-05-27

**Authors:** Cristiano P. Vieira, Taralyn M. McCarrel, Maria B. Grant

**Affiliations:** 1 Department of Ophthalmology and Visual Sciences, School of Medicine, University of Alabama at Birmingham, Birmingham, AL 35294, USA; cvieira@uabmc.edu; 2 College of Veterinary Medicine, University of Florida, Gainesville, FL 32611, USA; tmccarrel@ufl.edu

**Keywords:** mesenchymal stem cells, induced pluripotent stem cells, cell culture, automated cell system

## Abstract

Numerous studies demonstrate the essential role of mesenchymal stem cells (MSCs) in the treatment of metabolic and inflammatory diseases, as these cells are known to modulate humoral and cellular immune responses. In this manuscript, we efficiently present two novel approaches to obtain MSCs from equine or human sources. In our first approach, we used electro-acupuncture as previously described by our group to mobilize MSCs into the peripheral blood of horses. For equine MSC collection, culture, and expansion, we used the Miltenyi Biotec CliniMACS Prodigy system of automated cell manufacturing. Using this system, we were able to generate appoximately 100 MSC colonies that exhibit surface marker expression of CD105 (92%), CD90 (85%), and CD73 (88%) within seven days of blood collection. Our second approach utilized the iPSC embryoid bodies from healthy or diabetic subjects where the iPSCs were cultured in standard media (endothelial + mesoderm basal media). After 21 days, the cells were FACS sorted and exhibited surface marker expression of CD105, CD90, and CD73. Both the equine cells and the human iPSC-derived MSCs were able to differentiate into adipogenic, osteogenic, and chondrogenic lineages. Both methods described simple and highly efficient methods to produce cells with surface markers phenotypically considered as MSCs and may, in the future, facilitate rapid production of MSCs with therapeutic potential.

## 1. Introduction

Mesenchymal stem cells (MSCs) represent a versatile cell source for tissue regeneration [1] and remodeling due to their potent bioactivity, which includes modulation of inflammation, macrophage polarization toward pro-regenerative lineages, promotion of angiogenesis, and reduction in fibrosis. The paracrine signals of MSCs, their secretome, largely mediates their immunomodulatory and restorative potential [2]. There are close to 1000 clinical trials involving MSCs in the NIH clinical database (http://clinicaltrials.gov). A partial list of indications includes treatment strategies for type 2 diabetes and its complications, the repair of tendon injuries, arthritis, interstitial cystitis, systemic sclerosis, and Parkinson’s disease [3,4,5,6,7,8]. MSC-like populations have been generated from multiple sources, including bone marrow, adipose tissue, and umbilical cord blood [9]. However, considerable debate exists regarding the existence of MSCs in the peripheral blood [10,11,12], as their presence in the circulation is rare, ranging from 1 to 10^8^ peripheral blood MSCs [13].

We demonstrate that electro-acupuncture (EA) performed using the limb acupuncture sites LI-4 and LI-1I with GV-14 and GV-20, resulted in the mobilization of MSCs into the systemic circulation in rodents, horses, and humans [14,15]. EA promoted the central nervous system-dependent release of MSCs [15]. Peripheral blood derived-MSCs from EA demonstrated immunomodulatory potential and have been used in therapeutic applications in horses [14]. As peripheral blood represents a convenient source allowing rapid retrieval of cells for therapeutic indications, the current study sought to confirm the phenotype of EA-mobilized cells by flow cytometry and demonstrate the feasibility of using a GMP-like approach for their isolation and expansion. Thus, we tested the utility of EA-mobilized cells for ease of harvest and culture using CliniMACS Prodigy^®^, and used flow cytometry for rapid phenotypic characterization to define critical parameters needed for the successful translation to cell therapy. 

Obesity, metabolic disease, and diabetes afflict over 40% of the world’s population, and with these conditions comes a myriad of failed tissues and organs [16,17]. Diabetes can induce the replicative and stress-induced senescence of MSCs, thus limiting autologous MSCs as a cell therapy [18]. However, with the ability to genetically reprogram an individual’s somatic cells into human-induced pluripotent stem cells (hiPSCs) [19,20,21], autologous cell therapy may represent a viable option to regenerate and replace cells of various tissues and organs damaged by diabetes [22,23]. Thus, the second goal of this study was to develop a protocol to rapidly generate MSCs from hiPSCs mas mederived from diabetic and healthy subjects. We developed a protocol utilizing iPSC embryoid bodies (EBs). EBs are multicellular 3D aggregates formed from iPSCs that have three germ layer structures, recapitulate the beginning of embryonic development [24], and have high differentiation potential into new cell types [25,26]. 

In this study, we present novel methods for convenient retrieval using peripheral blood as the source of MSCs and rapid isolation, differentiation, and expansion of MSCs that lend themselves to clinical translation. Our findings indicate that peripheral blood containing EA-mobilized MSCs, when processed using an automated system, gave superior results in which a larger number of MSC colonies expressed CD105, CD73, and CD90 by 7 days, compared to 14 days with conventional culture conditions. We also found that harvesting and differentiating EBs from iPSCs with simplified culture media for 21 days resulted in the robust generation of MSCs from diabetic and healthy (nondiabetic) subjects with the same efficiency. Both equine and human MSCs were able to differentiate into osteogenic, chondrogenic, and adipogenic lineages. 

## 2. Results

### 2.1. CliniMACS Prodigy^®^ Isolates and Cultures Equine MSCs

EA was performed using the limb acupuncture sites LI-4 and LI-1I with GV-14 and GV-20 from three healthy horses [27]. Peripheral blood was obtained 3 h after the completion of the EA, a time previously optimized for the mobilization of MSCs. 

**Figure 1:**
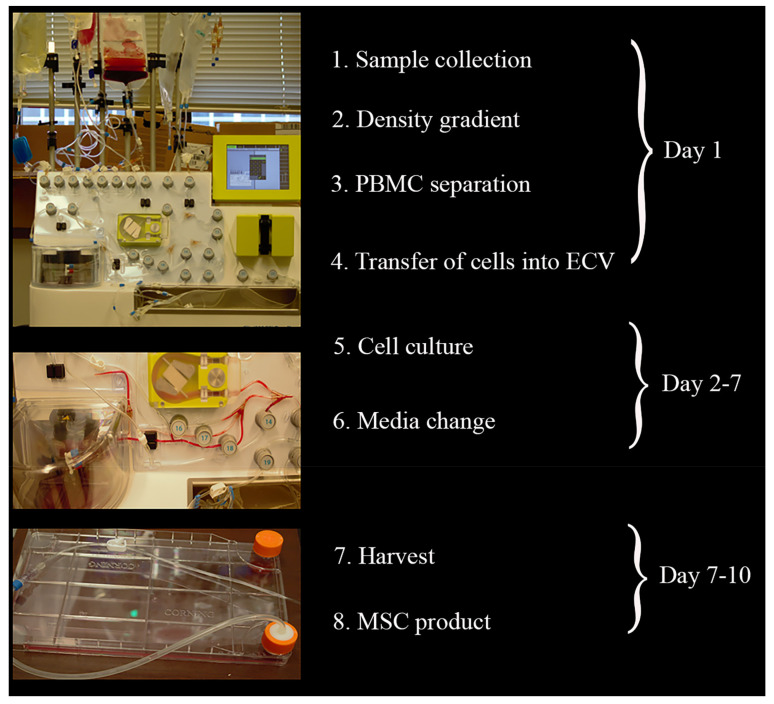
Schematic overview of adherent cell culture protocol using CliniMACS Prodigy^®^. Eight steps were enough to collect mononuclear cells, followed by cell culture and harvest of the mesenchymal stem cells. On the left side, photos of bags with buffer, cell culture media, and blood on the Prodigy system; transference of blood into the CentriCult Unit; and the CellSTACK^®^ used to seed the cells.

CliniMACS Prodigy^®^, a closed, automated cell processing system ensures standardized Good Manufacturing Practices (GMP)-compliant cell products. This study represented the first use of the Prodigy Adherent Cell Culture (AAC) system. Peripheral blood mononuclear cells were isolated by automated density gradient centrifugation of horse blood. For the AAC protocol, there was no need to remove erythrocytes. CliniMACS Prodigy^®^ modules were adjusted according to our protocol and can be seen in Figure 1. 

**Figure 2:**
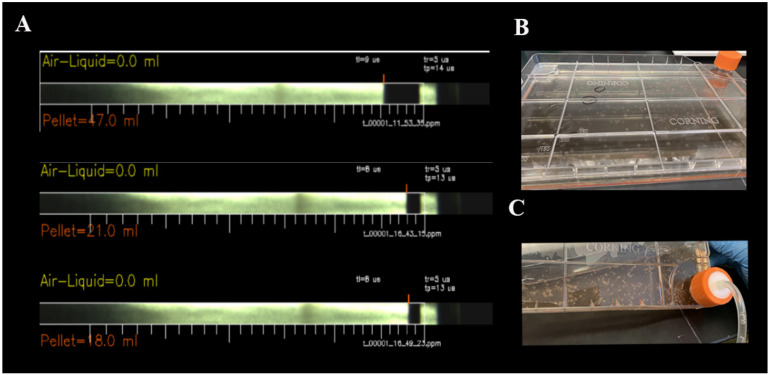
Pellet size and MSCs in the CliniMACS Prodigy^®^. (**A**) Interphase pictures of the pellet size on the CentriCult Unit after density gradient centrifugation. Three horses with 47, 21, and 18 mL of each differently sized pellet, respectively. (**B**) On day 7, mesenchymal stem cell colonies are visible in the CellSTACK^®^ in the external vessel. (**C**) Harvest of mesenchymal stem cells.

Cell pellets varied from 18.0 to 47.0 mL (Figure 2A) among the three horses. Prodigy concentrated the cells automatically and following separation, mononuclear cells were transferred into an external vessel, the Polystyrene CellSTACK^®^ with 636 cm^2^ cell growth area, connected to the machine. During this period, the cells stayed in the module culture with 5% CO_2_ at 37 °C. Details regarding the volumes of each reagent are provided in Table 1. 

**Figure 3:**
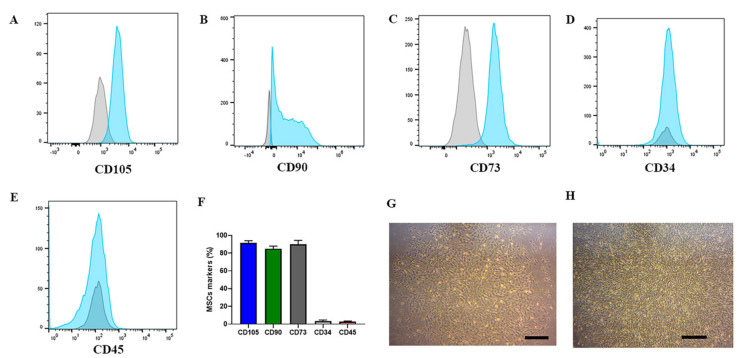
MSC phenotype on P0 in the CliniMACS Prodigy^®^. (**A**–**F**) expression of CD105^+^ (92%), CD90^+^ (85%), CD73^+^ (88%), CD34^+^ (3%), and CD45^+^ (< 2%). (**G**,**H**) the morphology of MSCs in the beginning of culture and with 90% of confluence, respectively. Bars = 20µm.

On days 4 and 6, cell culture media exchange occurred. By day 7, approximately 100 MSC colonies were observed (Figure 2B). On day 10, MSCs were harvested and flow cytometry was performed (Figure 2C). This was considered passage zero (P0). The phenotype of the MSCs was analyzed, and the P0 cells showed CD105^+^ (92%), CD90^+^ (85%), and CD73^+^ (88%). We also examined CD34^+^ cells (3%) and CD45^+^ cells (2%) (Figure 3A–F). MSC morphology is shown in Figure 3G,H.

### 2.2. MSC Phenotype Remains the Same after Changing to a Manual System

**Figure 4:**
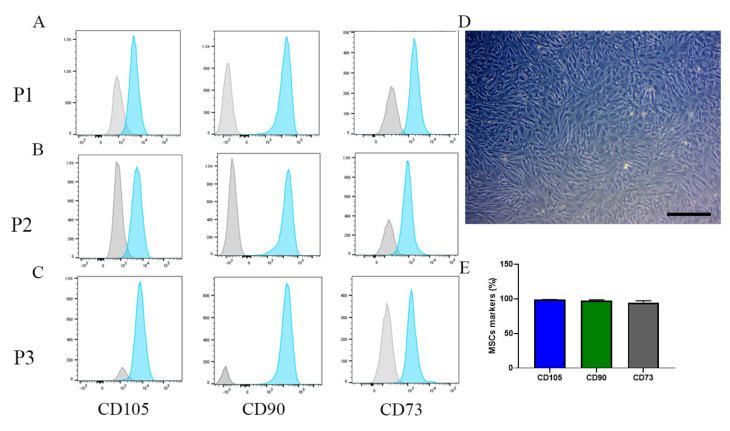
MSC phenotype on P1 to P3 in the manual system. (**A**) CD105^+^ expression in P1 (92%), P2 (95%), and P3 (98%). (**B**) CD90^+^ expression in P1 (87%), P2 (98%), and P3 (98%). (**C**) CD73^+^ expression in P1 (92%), P2 (95%), and P3 (95%). (**D**) the morphology of the MSCs in P3. Bar = 20µm; (**E**) MSC marker quantification from P3.

MSCs were expanded for three passages, and phenotypic characterization of the MSCs was performed. For these later passages, we used manual expansion (without Prodigy). Cells were harvested and seeded in a T75 flask at 2 × 10^6^ in standard media. In the following two days, 90% confluence was reached, and the cells were analyzed by flow cytometry. As expected, an increase in MSC markers was observed through progressive passages. P1, CD105^+^ (92%), CD90^+^ (87%), and CD73^+^ (92%) (Figure 4A); P2, CD105^+^ (95%), CD90^+^ (98%), and CD73^+^ (95%) (Figure 4B); and P3, CD105^+^ (98%), CD90^+^ (98%), and CD73^+^ (95%) (Figure 4C–E). 

### 2.3. EBs from iPSCs of Diabetic and Nondiabetic Subjects Can Differentiate into MSCs

**Figure 5:**
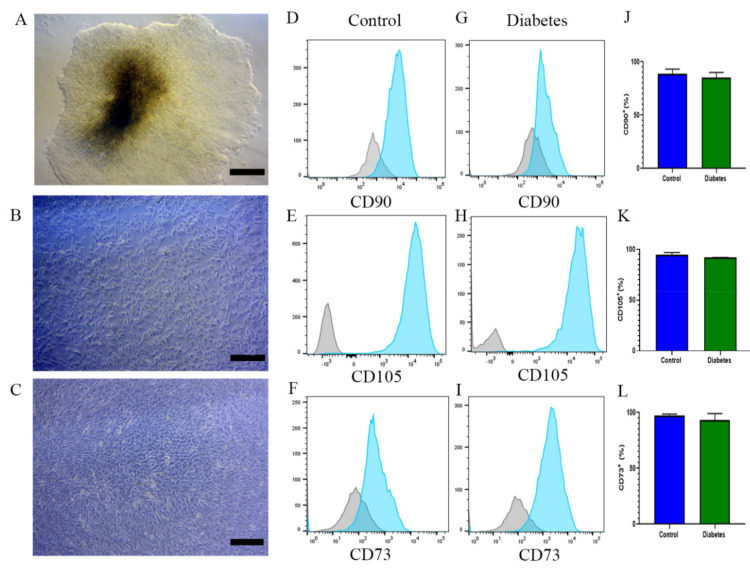
Embryoid bodies from the iPSCs of non-diabetic and diabetic subjects differentiate into MSCs. (**A**) EB morphology in a human iPSC colony. Bar = 50µm. (**B**,**C**) MSC morphology on day 21. Bar = 20µm. From a non-diabetic subject, (**D**) CD90^+^ (88%); (**E**) CD105^+^ (93%); and (**F**) CD73^+^ (98%). From diabetic subjects, (**G**) CD90^+^ (85%); (**H**) CD105^+^ (92%); and (**I**) CD73^+^ (93%). (**J**–**L**) quantification graphs comparing MSC markers of non-diabetic and diabetic subjects.

Mobilization of MSCs with EA is a strategy that is highly effective in healthy subjects. However, in the presence of chronic diseases such as diabetes, the utility of this approach has not been determined. Thus, alternative strategies are required to generate sufficient numbers of cells with robust function. With this objective, we sought to differentiate iPSCs of a diabetic origin into MSCs and phenotypically compare the cell product to the MSCs differentiated from the iPSCs of a nondiabetic/healthy origin. Embryoid bodies representing the three germinal layers can be found in a dense iPSC culture (Figure 5A). For this experiment, seven embryoid bodies of each nondiabetic or diabetic iPSC culture were seeded in 10 cm dishes coated with gelatin. On day 1, EBs were cultured in a 1:1 mTesrPlus and standard media. The following day, the media were replaced with standard media without mTesrPlus, and they were exchanged every two days until day 21 (Figure 5B,C). Flow cytometry analysis was performed and FACS sorted. The MSC population derived from diabetic and nondiabetic subjects were phenotypically similar. Nondiabetic MSCs exhibited CD105^+^ (93%), CD90^+^ (88%), and CD73^+^ (98%), whereas diabetic MSCs exhibited CD105^+^ (92%), CD90^+^ (85%), and CD73^+^ (93%) (Figure 5D–L).

### 2.4. Equine MSCs and iPSC-Derived MSC Showed Their Lineage Capacity of Differentiation

**Figure 6:**
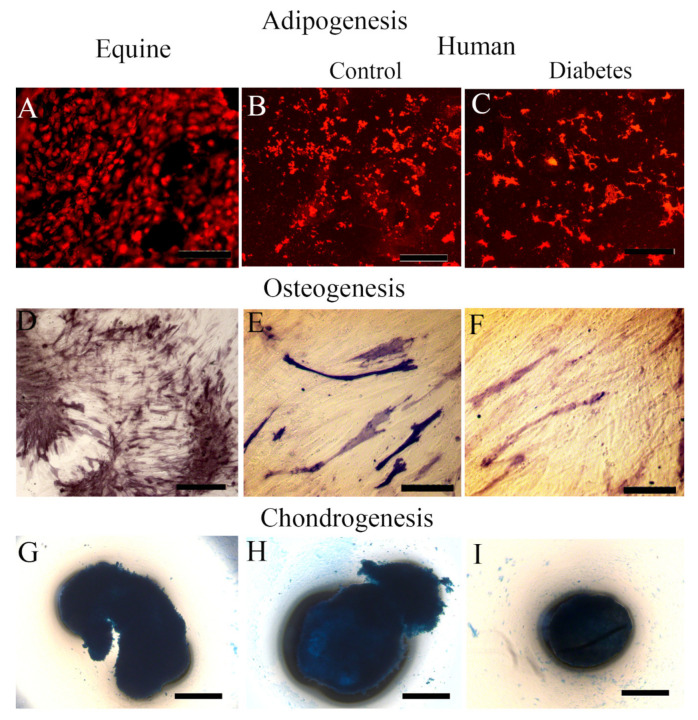
Lineage of differentiation ability of equine and human MSCs. (**A**–**C**): adipogenic differentiation of MSCs determined by Oil Red O staining in (**A**) (equine cells), (**B**,**C**) (control and diabetic human cells). Bar = 20µm. (**D**–**F**): osteogenic differentiation of MSCs determined by the detection of alkaline phosphatase activity in **C** (equine cells), (**D**,**E**) (control and diabetic human). Bar = 20µm. (**G**–**I**): chondrogenic differentiation of MSCs determined by Alcian blue staining in (**G**) (equine cluster), (**H**,**I**) (control and diabetic human clusters). Bar = 50µm.

In order to establish the full MSC potential of the equine and human MSCs, we verified their ability to differentiate into adipogenic, osteogenic, and chondrogenic lineages. Following being placed in adipogenic differentiation media, both equine MSCs and human iPSC-derived MSCs (diabetic and nondiabetic donors) showed robust ability to differentiate into adipocytes as determined by Oil Red O staining and fluorescence microscopy (Figure 6A–C). When placed under osteoblast differentiation conditions, equine MSCs (Figure 6D) showed a greater alkaline phosphatase activity in comparison to the human MSCs (Figure 6E,F). We have observed in the past that equine MSCs exhibit more rigorous osteoblast capacity compared to human MSCs (15). Equine and human MSCs both generated chondrocytes in three-dimensional clusters. After optimizing, using the differentiation medium and staining with Alcian blue, both equine and human cells generated chondrocyte “nodules” in micromass culture (Figure 6G–I).

## 3. Discussion

The large number of clinical trials found in the NIH database demonstrates that MSCs are well suited for regenerative therapies. MSC treatments are currently used for neurodegenerative diseases [7], kidney transplantation [6], liver disease [28], cartilage lesions, and osteoarthritis [8]. Previously, we advanced a safe and inexpensive strategy to mobilize MSCs from peripheral blood that involves EA at acupoints LI-4, and LI-11 with GV14 and GV-20. This strategy successfully mobilizes MSCs into the peripheral blood of horses [14], mice, rats, and humans [15] without the use of growth factors or pharmacological agents, and without any adverse side effects [14].

The purpose of this study was to establish an efficient and standardized protocol using peripheral blood for the isolation of MSCs and to maintain the viability and purity of MSCs in vitro while using GMP practices. The CliniMACS Prodigy^®^ platform has been used for T cells [29,30,31] and cancer immunotherapy studies [32]. The CliniMACS Prodigy^®^ platform offers many protocols for the collection, isolation, and culture of cells. In this study, we used the Adherent Cell Culture (AAC) system, which consists of the collection of mononuclear cells from the blood by density gradient centrifugation, inoculation into the external vessel, cell culture, media change, and harvest of the final product. 

Pre-processing is a step essential to prepare the Prodigy^®^ platform for all subsequent cell culture steps. The integrity test is a fundamental process that makes sure the machine is ready to receive the blood. The culture module controls the temperature and gas mix (CO_2_ and air) in the CentriCult Unit or the external vessel (ECV). The size of the CellSTACK^®^ to be used represents an important feature. MSCs are contact dependent for efficient proliferation and survival. Thus, based on the literature [14,15], cell density for plating was high. Culture conditions were standard, and all washes and media changes were entirely automated. On day seven, more than 100 MSCs colonies were detected and with similar morphology to that obtained by our previous manual approach. The MSC colonies were identified using CD90, CD105, and CD73 [33,34]. The percentage of CD34 and CD45 was less than 3%, which is not unexpected in MSC culture. The number of MSCs harvested was approximately 29 to 50 million cells in P0, which represents numbers that typically required more than 14 days to achieve using our previously published manual protocol. Interestingly, the purity of MSC markers increased with the number of passages reaching 95% in P3. Our study demonstrates that the automated cell system improves the purity, the number of cells isolated, and the efficiency of the expansion of equine MSCs compared to the standard manual protocol [14]. In this study, we confirm that the equine MSCs were able to differentiate into osteocytes, adipocytes, and chondrocytes. In passages P0 to P3, we established that equine MSCs express the characteristic MSC surface markers CD105^+^, CD90^+^, and CD73^+^.

In this study, we also describe a strategy utilizing embryoid bodies of iPSCs to obtain human MSCs. Guo and collaborators [25,26] reported that EBs are able to be cultured in organoid differentiation. Human iPSC cultures and EB formation improved the culture and differentiation efficiency of MSCs. Organoid establishment and early prediction of differentiation potential have been established previously [26]. The current studies expand on this published work by showing the feasibility of EB formation for iPSC cultures derived from diabetic and nondiabetic subjects. Zou and collaborators showed functional MSCs were applied to osteogenesis in 3D scaffolds from iPSCs without using EBs [35]. Our described protocol utilizes mTesrPlus solely to culture iPSCs following the harvest of EBs on day 10. This protocol is simpler than other protocols described in the literature [24,25,26] and achieved robust cell purity based on the classic MSC phenotype markers CD105, CD90, and CD73. Interestingly, the purity of the cells was comparable between the nondiabetic (88%) and diabetic subjects (85%). We confirmed that the human iPSC-derived MSCs were able to differentiate into osteocytes, adipocytes, and chondrocytes. The cells derived from diabetic donors exhibited the same robust ability to differentiate into all three lineages as the iPSCs that were derived from non-diabetic donors. In the future, iPSC-derived MSCs may become a therapeutic source for treating diabetes mellitus [3,4,20,21,36]. MSCs provide humoral and trophic support and can restore B-cell mass in type 1 diabetes and reduce insulin resistance in type 2 diabetes [5,37]. MSCs provide immunomodulatory behavior that could be beneficial for the treatment of vascular complications in diabetic subjects [38,39]. MSCs have shown therapeutic effects in diabetic subjects and facilitated the improvement of cutaneous healing by fostering proliferation and angiogenesis. MSCs also provide anti-inflammatory benefits by decreasing levels of IL-6, IL-1β, and TNFα and by increasing IL-10 levels to assist in motility/homing and improve cell survival [40,41]. MSCs express low levels of major histocompatibility complex (MHC) class I and no MHC class II, reducing the host immune response. Allogenic MSCs are well-tolerated and safe [5]. Diabetic iPSC-derived MSCs can represent an autologous treatment that is an excellent strategy to ameliorate chronic low-grade inflammation, which is typically seen with insulin resistance and β-cell dysfunction [5,37,38,39]. Clinical trials using MSC therapy show a benefit in patients by improving glycemia [5]. Studies are underway to utilize the CliniMACS Prodigy^®^ system to isolate and expand human MSCs obtained from peripheral blood.

In summary, we provide two approaches to enhance the clinical utility of MSCs in this study. First, we show that the isolation and culture of MSCs can be improved over standard approaches by employing the CliniMACS Prodigy^®^ system. Moreover, this provides a GMP-like approach and an automated system. Second, we demonstrate that EBs can be generated from iPSCs derived from both healthy and diabetic donors and can likely be exploited to generate highly purified and robust MSCs from individuals with longstanding diabetes. Nonsurgical and non-pharmacological methods for MSC mobilization, isolation, and expansion are needed, as are strategies that facilitate the generation of MSCs from individuals with debilitating and chronic diseases. 

## 4. Material and Methods

### 4.1. Experimental Protocols

All experimental protocols involving EA to mobilize MSCs into the peripheral blood of horses were approved by the Institutional Animal Care and Use Committee (IACUC) protocol #201207468 at the University of Florida. For the human samples, peripheral blood samples from diabetic subjects and age- and gender-matched healthy individuals were collected at the UAB Callahan Eye clinic under the institutional review board-approved protocol (IRB-300000173) at the University of Alabama at Birmingham (UAB).

### 4.2. Electro-Acupuncture Procedure

Horses received electro-acupuncture at acupoints LI-4 and LI-11 with GV-14 and GV-20. Points were stimulated with a frequency of 20 Hz for 45 min using an electro-acupuncture instrument (JM-2A model, Wuxi Jiajian Medical Instrument, Inc., Wuxi, China) [14,27]. The EA procedure started at 9:00 a.m., and 100 mL of blood was collected 3 h later in ethylenediaminetetraacetic acid (EDTA)-containing tubes. Peripheral blood was maintained at room temperature (~25 °C) until processing within the CliniMACS Prodigy^®^.

### 4.3. Adherent Cell Culture Protocol Using CliniMACS Prodigy^®^

Equine MSC isolation utilized the Adherent Cell Culture system (Figure 1) from Miltenyi Biotec CliniMACS Prodigy^®^ where detailed protocols can be found (http://miltenyibiotec.com Accessed on: 11 May 2021). Briefly, EDTA tubes containing peripheral blood were transferred to 100 mL bags using a sterile welder. Transfer of the cellular starting material into the chamber of the CliniMACS Prodigy TS730^®^ was performed next. In the chamber, equine blood was washed with a CliniMACS^®^ buffer that contained 0.5% FBS and was underlaid with Ficoll-Paque Plus (GE Healthcare Bio-sciences, Pittsburgh, Pennsylvania, USA), followed by centrifugation. Interphase pictures were taken of the pellet by the microscope of the chamber. The remaining material in the chamber was discarded, and the chamber was then cleaned with buffer to avoid carry-over of platelets and erythrocytes. No coating parameters were added to the external vessel, the Polystyrene CellSTACK^®^, as our protocol does not require this. The cell suspension was transferred to the CentriCult Unit, followed by washing and addition of standard media. Media consisted of a 1:1 mix of EBM-2 (Lonza, Walkersville, MD, USA) and low-glucose MEM alpha (Lonza) medium and 1% antibiotics/antimycotics ented with 15% FBS (Gibco, Carlsbad, CA, USA). The culture mode offers the possibility of the culture of cells in the chamber or in the external vessels. The parameters of cell culture are 37 °C and 5% CO_2_.

The media exchange module replaced the existing culture medium with new media. In order to access this module, the cell culture module was interrupted. Fresh cell culture media were suspended in a bag and pre-warmed using the heat exchange cartridge of the CliniMACS Prodigy TS730^®^. All of the valves that connect the external vessel to the machine were opened, and the media change module was activated. The existing medium was aspirated and replaced with 130 mL of fresh regular media. After the media were changed, the cell culture module was activated. When the MSC colonies were visible on day 7, the harvest module was initiated, and 50 mL of TrypLE^TM^ Express Enzyme (Gibco, Grand Island, NY, USA) was added to the external vessel plate for 5 min. Regular media were added to the plate to inactivate the dissociation buffer. The entire cell suspension was transferred into a new bag and was ready for culture. At this point, the cell material suspension was taken out from the Prodigy platform for subsequent experiments.

### 4.4. Cell Culture 

After the cell suspension was removed from the Prodigy, the cell culture was subsequently performed manually. From passage 1 to passage 3, 2 × 10^6^ MSCs were seeded into the T75 flask (Thermofisher Scientific, Rochester, NY, USA) in regular media, and media change was performed every other day. When the confluence reached ~90%, the cells were harvested and flow cytometry analysis was performed.

### 4.5. hiPSC Generation and Culture

Human PBMC were stored in liquid nitrogen and shipped to ALSTEM, INC, (Richmond, CA, USA) for reprogramming into hiPSCs and for characterization. iPSCs were maintained in mTesrPlus media (STEMCELL Technologies, Vancouver, BC, Canada) on Matrigel (STEMCELL Technologies, Vancouver, Canada) in a 37 °C and 5% CO_2_ incubator. Cell culture media were changed every day, and cells were examined for the elimination of differentiated cells. After 10 days, large colonies had an embryoid body in their central region. Seven of the EBs were seeded in a new dish coated with gelatin in 1:1 mTESRplus media and regular media overnight. From day 2 to day 21, the media were changed every other day with regular media only. On day 21, phenotypic markers of MSCs were assessed by flow cytometry and by morphology on a phase-contrast microscope.

### 4.6. Phenotypic Characterization of Equine and Human MSCs

The MSC phenotype proposed by the International Society for Cell Therapy was utilized in this study (33). Cells were washed in PBS containing 2% FBS and 1% EDTA (staining buffer) and then re-suspended in 100 μL of staining buffer. The following antibodies were used: CD105-FITC (Invitrogen, Carlsbad, CA, USA, cat# MA-1-80943), CD73-PE-Cy7 (BD Biosciences, San Jose, CA, USA cat# 561258), CD90-PerCP-Cy5.5 (BD Biosciences, San Jose, CA, USA, cat# 561557), CD34-PE (Novus Biologicals, Centernnial, CO, USA, cat# 070920), and CD45-AF700 (Southern Biotech, Birmingham, AL, USA, cat #9625-27). The samples were incubated for 30 min in the dark at room temperature and then washed 2 times with 2 mL of staining buffer and analyzed by flow cytometry (BD Celesta, CA, USA). The data were evaluated using FlowJo v10 software (Becton, Dickinson, and Company; 2019).

### 4.7. Adipogenic, Osteogenic, and Chondrogenic Differentiation of Equine and Human iPSC-Derived MSCs

The differentiation of equine and human MSCs into adipocytes, osteocytes, and chondrocytes was performed using the mesenchymal stem cell assays developed by Miltenyi Biotec. For adipogenic differentiation, StemMACS AdipoDiff Media (Miltenyi, Auburn, CA, USA 130-091-677) was used, and cells were stained with Oil Red O (Millipore Sigma, ST Louis, MO, USA cat# 0975525G). For osteogenic differentiation, StemMACS OsteoDiff Media (Miltenyi, Auburn, CA, USA, 130-091-678) was used, followed by the detection of alkaline phosphatase activity using SIGMAFAST™ BCIP^®^/NBT (Sigma-Aldrich, ST Louis, MO, USA, cat# B5655-25TAB). For chondrogenic differentiation, StemMACS™ ChondroDiff Media (Miltenyi, Auburn, CA, USA, 130-091-679) was used, followed by Alcian blue (Millipore Sigma, ST Louis, MO, USA, cat# TMS010C) staining of chondrocyte “nodules”. MSCs from three separate horses and MSCs from two diabetic and two control human subjects were used in duplicate. 

## Figures and Tables

**Table 1:** Reagents and supplies used for one run in the CliniMACS Prodigy.

Supply	Quantity	Catalog No.	Company
TS 730	1 unit	170-076-603	Miltenyi Biotec
Transfer bag	3 units	T3108	Chartermedical
Cell Stack	1 unit	3268	Corning
1 m tubing extension	1 unit	170-076-606	Miltenyi Biotec
CliniMACS buffer	3 L	130-070-525	Miltenyi Biotec
Ficoll-Paque	145 mL	17-1440-03	GE Healthcare Bio Sciences
FBS heat inactivated	300 ml	10-082-147	Gibco
Tryple Express	100 mL	1260013	Gibco
EBM	750 mL	CC3156	Lonza Walkersville
MEM	750 mL	10009CV	Corning
Penicillin–streptomycin–glutamine	20 mL	10378016	Gibco

## Data Availability

The data presented in this study are available on request from the corresponding author.
